# MicroRNA Regulation of Glycolytic Metabolism in Glioblastoma

**DOI:** 10.1155/2017/9157370

**Published:** 2017-07-19

**Authors:** Huda Alfardus, Alan McIntyre, Stuart Smith

**Affiliations:** Children's Brain Tumour Research Centre, Queen's Medical Centre, D22 Medical School, School of Medicine, University of Nottingham, Nottingham NG7 2UH, UK

## Abstract

Glioblastoma (GBM) is the most aggressive and common malignant brain tumour in adults. A well-known hallmark of GMB and many other tumours is aerobic glycolysis. MicroRNAs (miRNAs) are a class of short nonprotein coding sequences that exert posttranscriptional controls on gene expression and represent critical regulators of aerobic glycolysis in GBM. In GBM, miRNAs regulate the expression of glycolytic genes directly and via the regulation of metabolism-associated tumour suppressors and oncogenic signalling pathways. This review aims to establish links between miRNAs expression levels, the expression of GBM glycolytic regulatory genes, and the malignant progression and prognosis of GBM. In this review, the involvement of 25 miRNAs in the regulation of glycolytic metabolism of GBM is discussed. Seven of these miRNAs have been shown to regulate glycolytic metabolism in other tumour types. Further eight miRNAs, which are differentially expressed in GBM, have also been reported to regulate glycolytic metabolism in other cancer types. Thus, these miRNAs could serve as potential glycolytic regulators in GBM but will require functional validation. As such, the characterisation of these molecular and metabolic signatures in GBM can facilitate a better understanding of the molecular pathogenesis of this disease.

## 1. Introduction

Glioblastoma (GBM) is the most common malignant primary brain tumour in adults, accounting for 12–15% of all intracranial tumours [1]. GBM is also the most aggressive form (World Health Organization (WHO) grade IV) of glioma, an umbrella term for tumours thought to originate from glial progenitors such as astrocytoma [2, 3]. GBM can develop de novo or through the progression of preexisting low WHO grade glioma (WHO grade II, diffuse astrocytoma) that develop into high WHO grade glioma (WHO grade III, anaplastic astrocytoma) and GBM [3]. In general, GBM shows an increased incidence in Caucasian populations [4]. In the UK and the United States alone, the annual GBM incidence rate ranges between 4.64 and 5.26 per 100,000 people [5, 6]. The current GBM treatment standards consist of maximal surgical resection followed by radiotherapy with concurrent temozolomide (TMZ) chemotherapy, followed by six cycles of maintenance TMZ chemotherapy [7, 8]. However, GBM prognosis remains poor with a median overall survival of 14 months and a 5-year survival rate of less than 10% [9, 10].

Metabolic reprogramming is appreciated as important hallmarks of cancer, including GBM, and it serves as a valuable therapeutic target [11, 12]. Many cancers seem to employ aerobic glycolysis as their metabolic programme of choice to fulfil their bioenergetic and anabolic requirements for rapid growth and enhance their survival in response to microenvironmental stress [11–15]. GBM is characterised by increased aerobic glycolysis compared to normal brain, which may contribute to the malignant progression of GBM [16, 17]. Aerobic glycolysis, also known as the Warburg effect, is a catabolic process that, in the presence of oxygen, converts one glucose molecule into two lactate molecules [18]. Aerobic glycolysis is controlled by tumour suppressors and oncogenic signalling pathways in both tumour and normal cell [19]. Aberrant expression of oncogenes and tumour suppressor genes in GBM alters the expression and activity of glycolytic transporters and metabolic enzymes. The expression of these metabolic and regulatory genes is modulated by a class of small nonprotein coding RNAs, called microRNAs (miRNAs), that regulate gene expression at the posttranscriptional level [20]. To date, great advances have been made to understand the role of miRNAs in the regulation of glycolytic metabolism in GBM. This review aims to establish links between miRNAs expression levels, the expression of GBM glycolytic regulators and the malignant progression and prognosis of GBM. First, the review will discuss the role of miRNAs in regulating GBM glycolytic metabolism by directly targeting glycolytic genes and via the regulation of tumour suppressors and oncogenic signalling pathways. Some of these miRNAs have also been shown to regulate glycolytic metabolism in other tumours. The review will then present differentially expressed miRNAs in GBM which have been reported to be involved in the regulation of glycolytic metabolism in other tumours. Potentially, these miRNAs could also have a glycolytic regulatory role in GBM, but that is yet to be experimentally validated. As such, the characterisation of these molecular and metabolic signatures in GBM can facilitate a better understanding of the molecular pathogenesis of this disease.

## 2. Regulation of Glycolytic Metabolism by Tumour Suppressors and Oncogenic Signalling Pathways in GBM

The dysregulation of tumour suppressors and oncogenic signalling pathways plays an important role in determining the glycolytic phenotype of GBM. Comprehensive genomic characterisation [21] using 206 GBM samples performed by The Cancer Genome Atlas (TCGA) Network showed that genetic alterations are frequently found within the receptor tyrosine kinases (RTKs) and their downstream effector pathways. Using 91 GBM samples, it was shown that the RTKs, hepatocyte growth factor receptor (encoded by* c-Met*) and platelet-derived growth factor receptor-*α* (PDGFRA), are aberrantly activated in 4% and 13% of GBM cases, respectively [21]. However, gain-of-function mutations and/or amplification in the epidermal growth factor receptor* (EGFR)* are the most common in GBM (45% of GBM cases) [21]. Active EGFR signals via multiple effector pathways including* RAS* and phosphatidylinositol 3-kinase* (PI3K)* signalling cascades.

The cytoplasmic domain of EGFR recruits adaptor proteins to activate RAS [22]. Moreover, the activation of RAS signalling can be achieved through losing the expression of the RAS antagonist, neurofibromin 1 (NF1), which is observed in about 14% of GBM cases [21]. RAS activates PI3K while PI3K can independently be activated by the cytoplasmic domain of EGFR [23, 24]. PI3K is aberrantly activated in 15% of GBM cases [21]. Activated PI3K catalyses the phosphorylation of phosphatidylinositol (4,5)-bisphosphate (PIP2) into phosphatidylinositol (3,4,5)-trisphosphate (PIP3) [25], which can be reversed by the phosphatase tensin and homologue [26] (PTEN; homozygous deletions and mutations are found in 36% of GBM cases) [21]. Following its recruitment to the plasma membrane by PIP3, protein kinase B (Akt) is phosphorylated by 3-phosphoinositide-dependent protein kinase 1 (PDK1) [27, 28]. Akt is found to be amplified in 2% of GBM cases [21]. Activated Akt activates both the rapamycin sensitive mTOR complex 1 (mTORC1) and the rapamycin insensitive mTOR complex 2 (mTORC2). First, Akt phosphorylates the SIN1 subunit of mTORC2 and, thus, induces the activation of mTORC2. In a positive feedback loop, mTORC2 phosphorylates and thereby fully activates Akt [29]. Second, Akt phosphorylates and inhibits TSC2 thereby relieving the inhibitory effects of the TSC1-TSC2 complex on mTORC1 [30–32]. mTORC1 is also negatively regulated by the energy-sensing AMP-activated protein kinase (AMPK). The reduction in ATP causes an increase in the AMP : ATP ratio leading to the activation of AMPK [33, 34]. AMPK mediates an activating phosphorylation of TSC2 and an inhibitory phosphorylation of the mTORC1 subunit Raptor [35, 36]. In GBM, the activation of mTOR signalling cascade leads to the upregulation of transcription factors such* c-Myc *[37] which upregulate the expression of glycolytic genes [38, 39].

In addition, Akt promotes GBM glycolytic phenotype by increasing the expression and membrane translocation of glucose transporters 1 and 3 (GLUT1 and GLUT3) which are upregulated in GBM [40, 41]. Akt also regulates glycolysis by enhancing the activity and the cellular localisation of hexokinase II (HKII), which phosphorylates glucose in the first step of glycolysis [42]. The role of* Akt* in GBM aerobic glycolysis was supported by Elstrom et al. (2004) [43] who observed differences in the glycolytic rates of various GBM cell lines which were then attributed to the differences in Akt activity levels in these cells. In their study, two GBM cell lines were grown in normal glucose conditions; LN18 cells with constitutive Akt activity, as measured by Akt phosphorylation, showed higher rates of aerobic glycolysis than LN229 cells with low Akt activity. The inhibition of the upstream regulator, PI3K, abolished Akt phosphorylation and reduced the glycolytic rate of LN18 cells while the overexpression of Akt in LN299 cells was sufficient to stimulate high rate of glycolysis [43]. This suggests that the PI3K/Akt pathway is a key glycolytic regulator in GBM.

## 3. Clinical Stratification of GBM in relation to the Expression of Glycolytic Genes

Recently, the presence of heterozygous gain-of-function mutations within the active site of isocitrate dehydrogenase-1* (IDH1)*, a Krebs cycle enzyme that reduces *α*-ketoglutarate into 2-hydroxyglutarate (2-HG), has been associated with improved clinical outcomes in GBM [44–46]. Mutant IDH1 produces increased levels of 2-HG which inhibit histone demethylating enzymes, thereby, leading to extensive DNA methylation of CpG islands within multiple promoter regions across a large number of loci (CpG island methylator phenotype) [46–49]. The promotor methylation of the O6-Methylguanine-DNA Methyltransferase* (MGMT)* gene, encoding a DNA repair protein that can confer resistance to the alkylating chemotherapeutic agent TMZ by reversing mutagenic O6-alkyl-guanine back to guanine [50, 51], causes transcriptional silencing of the gene. Hence, patients with* MGMT* methylation show improved response to TMZ [52–56].

*IDH1* status can also distinguish different glycolytic phenotypes of GBM, which may contribute to the different clinical behaviour of tumours with and without the* IDH1* mutations [57]. In* IDH1 *mutant GBMs, the expression of 3 glycolytic enzymes, glucose-1-dehydrogenase, enolase 1, and lactate dehydrogenase isoform A* (LDHA)*, is downregulated while the expression of 2 other glycolytic enzymes, fructose-bisphosphate C and lactate dehydrogenase isoform B* (LDHB)*, is upregulated compared to* IDH1 *wild-type GBMs [58, 59]. Both LDHA and LDHB can convert pyruvate to lactate; however, LDHB is thought to be more sensitive to substrate inhibition by pyruvate [60]. As such, intracellular lactate levels are reduced in* IDH1* mutant compared to* IDH1* wild-type GBMs, suggesting that* IDH1 *mutation is associated with mitigated aerobic glycolysis in* IDH1* mutant GBMs [61].

**Figure 1:**
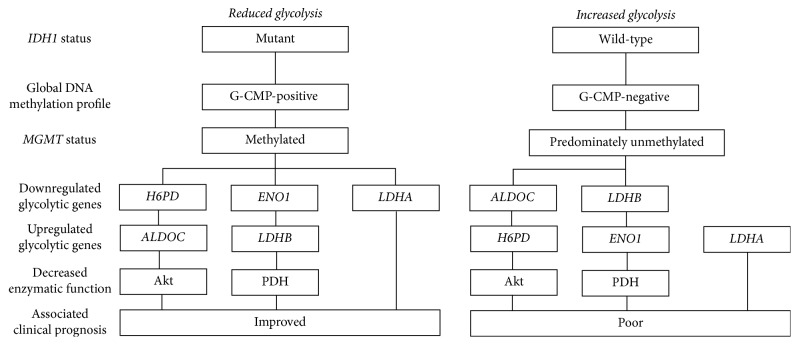
*Clinical classifications and glycolytic phenotype of GBMs*. H6PD: glucose-1-dehydrogenase, ENO1: enolase 1, LDHA/B: lactate dehydrogenase isoform A/B, ALDOC: fructose-bisphosphate C, and PDH: pyruvate dehydrogenase.

In addition, the activity of pyruvate dehydrogenase (PDH), which is involved in the conversion of pyruvate into Acetyl-CoA, was found to be decreased in* IDH1* mutant compared to* IDH1 *wild-type GBMs [62]. In* IDH1* mutant cell, 2-HG can induce high expression of pyruvate dehydrogenase kinase-3 which mediates an inhibitory phosphorylation of PDH that results in reduced PDH activity [62].* IDH1* mutant GBMs also show decreased Akt phosphorylation and downregulation in the expression of genes that are regulated by the* PI3K/Akt* pathway when compared with GBMs that lack* IDH1* mutation [63]. Thus, GBMs with* IDH* mutations show a relatively reduced glycolytic phenotype compared to GBMs with wild-type* IDH* (Figure 1).

## 4. miRNAs in GBM

Besides acting as biomarkers [64], miRNAs are involved in the regulation of diverse cellular functions in GBM, including cell death, migration, invasion, proliferation, drug resistance, and angiogenesis (recently reviewed in [65]). Moreover, miRNAs can also act as critical regulators of glycolytic metabolism in GBM, which this review will focus on in more detail. There are two ways by which miRNAs regulate GBM glycolytic metabolism. Firstly, miRNAs can directly regulate the expression of genes taking part in glucose uptake and glucose metabolism in GBM, miR-106a regulates* GLUT3* [66], miR-143 regulate* HKII *[67], and let-7-a and miR-326 regulate* PKM2* [34, 68], which will be detailed in Section 5. Secondly, miRNAs can also regulate glycolysis indirectly by regulating the signal transduction of RTKs via PI3K/AKT and RAS pathways which leads to the upregulation of* c-Myc* expression and Akt activity, both of which enhance the expression and the function of glycolytic transporters and enzymes [37, 40–42]. Hence, the dysregulation of metabolic regulatory signalling pathways by miRNAs can additionally contribute to the upregulation of glycolysis in GBM. In Section 6, the role of 21 miRNAs in the regulation of 14 components of the RTKs effector pathways will be discussed.

Furthermore, the expression of glycolytic regulatory miRNAs is sometimes associated with the malignancy grade of glioma and the prognosis of GBM, as will be described in the next two sections. Only studies that have validated the direct mRNA-miRNA interaction via luciferase assay were included in this review. However, the mRNA-miRNA interactions were studied in unstratified GBM patient cohort, where* IDH1* status was not reported. Thus, it must be noted that* IDH1 *status may confound many reported associations between expression of particular miRNAs and their target gene and the malignant progression and prognosis of GBM. In addition, many prognostic associations for the specific miRNAs discussed in this review are only based on retrospective analyses of patient cohorts of various sizes, in which the treatment regime might not have been according to current standard of care which may limit the generalisation of such findings.

## 5. miRNA Regulation of Glycolytic Transporters and Enzymes in GBM

**Figure 2:**
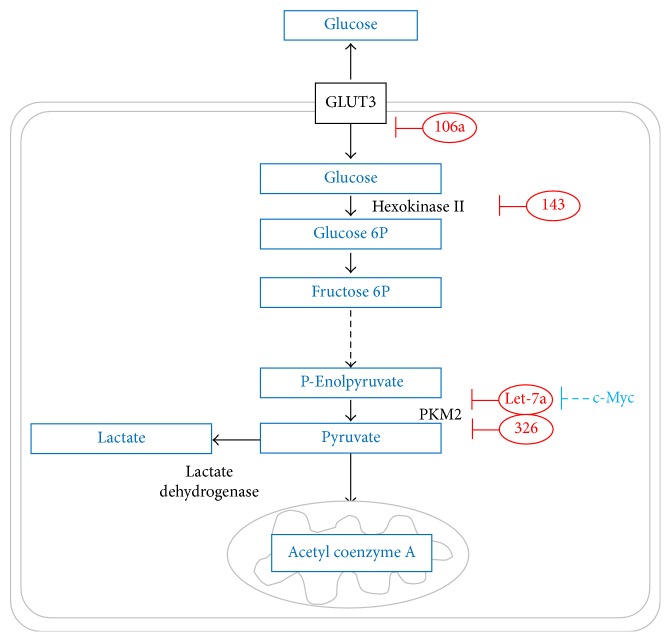
miRNA regulation of glycolytic transporters and enzymes in GBM. Red ovals represent downregulated miRNAs and blunt ends designate negative regulation. c-Myc (downstream effector of RTKs signalling pathways) upregulates glycolysis by negatively regulating let-7a which targets PKM2: pyruvate kinase type M2. Dashed black lines indicate that several steps have been omitted. Mitochondria represent entry into the citric acid cycle. P: phosphate. P-: phospho-.

To date, four miRNAs have been reported to directly modulate the expression of glycolytic transporters and enzymes in GBM (Figure 2). The expression of GLUT3 is downregulated by miR-106a [66]. However, miR-106a is found to be downregulated in GBM compared to normal brain [66]. The low miR-106a expression is associated with shorter-term survival of GBM patients [66, 69, 70]. Moreover, the expression of miR-106a in high WHO grade glioma is lower than that in low WHO grade glioma, an expression pattern that is opposite to GLUT3 [41, 66, 71]. Thus, miR-106a downregulation promotes glycolysis and enhances glucose flux by releasing the miRNA-mediated suppression on GLUT3.

Furthermore, the glycolytic enzyme* HKII *is targeted by miR-143 [67] which is also found to be downregulated in GBM compared to low WHO grade glioma and normal brain [67, 72]. miR-143 expression is negatively correlated with HKII levels [67], which is associated with poor prognosis [73]. Another glycolytic enzyme,* PKM2*, is regulated by the miRNA, let-7a [68]. PKM2 is the M2 isoform of pyruvate kinase (PK), the terminal glycolytic enzyme which converts phosphoenolpyruvate to pyruvate [74]. PKM2 has a relatively decreased enzymatic activity which leads to the accumulation of upstream glycolytic intermediates that can be channelled into the biosynthetic pathways [75]. PKM2 is selectively expressed at low levels in GBM but is completely absent in normal brain [34].* c-Myc*, which is also targeted by let-7a, upregulates the expression of the heterogeneous nuclear ribonucleoprotein A1* (hnRNPA1)* splicing factor which, in turn, downregulates let-7a in a positive feedback loop [68]. hnRNPA1 binds to the pri-let-7a and blocks its processing by Drosha [76]. In addition, hnRNPA1 mediates the splicing of PK into the PKM2 isoform as well as that of the Myc-interacting partner Max into the Delta Max isoform. Delta Max forms a complex with c-Myc to drive the transcription of the c-Myc target genes, including* hnRNPA1 *[77–80]. As such, let-7a/c-Myc/hnRNPA1/PKM2 regulatory loop ensures the downregulation of let-7a in order for* PKM2* to be expressed in GBM. Another miRNA which targets* PKM2*, miR-326, is downregulated in GBM compared to normal brain as a result of the decreased transcription of its host gene, *β-arrestin 1 *[34, 81]. In GBM cells, the overexpression of miR-326 or the knockdown of its target, PKM2, reduced cellular proliferation, metabolic activity, and ATP levels [34]. Such decrease in ATP levels was, however, rescued by transfecting GBM cells with PKM2 mRNA lacking the 3′-UTR which renders them insensitive to miR-326 [34]. Therefore, miR-326 mediates its effects on tumour metabolism by repressing* PKM2* expression.

## 6. miRNA Regulation of RTKs and Their Downstream Effector Pathways in GBM

**Figure 3:**
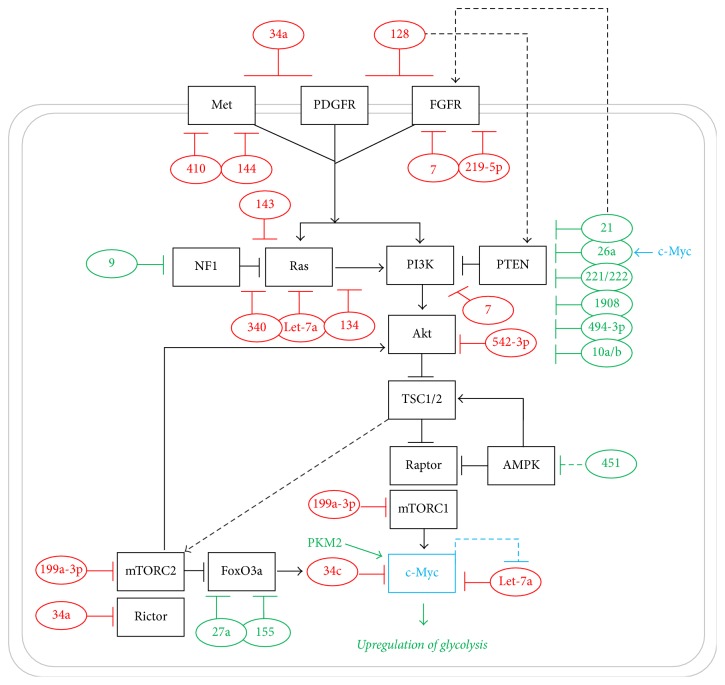
miRNA regulation of glycolytic regulatory signalling pathways in GBM. Arrowheads designate positive regulation. Blunt ends designate negative regulation. Dashed lines indicate indirect effects. Green and red ovals indicate upregulated and downregulated miRNAs. c-Myc regulates miRNAs and is regulated by the glycolytic enzymes PKM2: pyruvate kinase type M2.

Multiple components of the RTKs effector pathways are tightly regulated by miRNAs (Figure 3, details of the interactions between the different components have been described in Section 2). In the context of cancer, miRNAs that downregulate tumour suppressors are called oncomiRs while miRNAs that target and suppress oncogenes are called tumour suppressor miRNAs. As such, oncomiRs and tumour suppressor miRNAs are found to be overexpressed and downregulated in cancer cells, respectively [82, 83]. As such, miRNAs that suppress glycolytic metabolism by targeting oncogenic components of the RTKs, which are the tumour suppressor miRNAs, are downregulated while those that promote glycolysis by targeting metabolic tumour suppressor genes, which are the oncomiRs, are upregulated in GBM as discussed below. Furthermore, the expression levels of these miRNAs either (i) are invariant across the different glioma WHO grades, suggesting that the expression change of a particular miRNA might signify a key early event in gliomagenesis, or (ii) can distinguish different glioma WHO grades, thereby serving as a potential biomarkers of glioma progression [84, 85].

### 6.1. miRNA Regulation of RTKs

Three RTKs (*c-Met, PDGFRA*, and* EGFR*) have been reported to be targeted by miRNAs which are found to be downregulated in GBM in order to enable the upregulation of the downstream signalling which may, in turn, promote glycolytic metabolism, although it has not directly been shown.* c-Met* is a target of miR-410, which is downregulated in GBM compared to low WHO grade glioma and normal brain [86].* c-Met *is also targeted by miR-144-3p which is downregulated in GBM [87]. miR-144-3p expression is inversely correlated with glioma WHO grade and overall patient survival [87]. The expression of miR-34a, another negative regulator of* c-Met,* is also inversely correlated with glioma WHO grade [88–91]. Moreover, miR-34a expression in GBM is suppressed by* PDGFRA*, which is targeted by miR-34a in a negative feedback loop [88]. The administration of imatinib, an inhibitor developed for BCR-ABL which can also inhibit PDGFRA [92, 93], reversed the negative effect of PDGFRA on miR-34a expression [88]. Furthermore, miR-128, which targets* PDGFRA* and* EGFR *[94], is downregulated in GBM relative to low WHO grade glioma [95–99].* EGFR* is also targeted by miR-219-5p, which is downregulated in GBM [100, 101]. In addition,* EGFR* is indirectly regulated by miR-21 which targets the* EGFR* transcriptional activator* STAT3* [102–105]. The expression of miR-21 is positively correlated with glioma WHO grade and decreased patient survival [100, 105–118]. Further links between miRNA and* RTKs* expression in GBM were suggested by Kefas et al. (2008) and Webster et al. (2009) who proposed that* EGFR* is targeted by miR-7 [119, 120]. miR-7 shows a brain-specific expression; however, miR-7 shows a relatively decreased expression in GBM [121]. Although, pri-miR-7 levels are similar in both GBM and normal brain, pre-miR-7 levels are decreased in GBM. This suggests that changes of regulatory mechanisms that control the processing of pri-miR-7 to pre-miR-7 could be responsible for the decrease in miR-7 expression in GBM [119].

### 6.2. miRNA Regulation of* RAS*

One of the effectors of the RTKs signalling is the RAS pathway. RAS is antagonised by the tumour suppressor NF1 which is regulated by miR-9 [122]. miR-9 is upregulated in GBM, which releases the NF1-mediated suppression on RAS. miR-9 upregulation is associated with poor prognosis in GBM [122, 123]. Furthermore,* RAS* (specifically* N-RAS*) is regulated by miR-143 [72], which also targets the glycolytic enzyme* HKII *[67], and by miR-340, which is downregulated in GBM and is associated with poor prognosis [124, 125]. The expression of another RAS gene* (K-RAS)* is regulated by let-7a [126], which also regulates both the glycolytic enzyme* PKM2* and the glycolytic driver* c-Myc* [68].* K-RAS* is further regulated by miR-134, which is found to be downregulated in GBM [127]. The regulation of* RAS* by multiple miRNAs that directly target glycolytic enzymes could indicate a strong link between* RAS* expression and enhanced glycolysis in GBM, yet to be investigated.

### 6.3. miRNA Regulation of* PI3K/Akt*

Another downstream effector of RTKs signalling, which is upregulated in GBM and may thus be contributing to the enhanced GBM glycolytic metabolism, is the* PI3K/Akt* pathway. PI3K is directly regulated by miR-7, which also regulates* EGFR *as mentioned above [121]. The overexpression of miR-7 was shown to downregulate* PI3K* expression in a dose-dependent fashion [121]. Another EGFR regulator, miR-21, regulates the expression of the tumour suppressor and the PI3K antagonist,* PTEN* [105]. miR-21 in GBM targets and downregulates* PTEN* while the knockdown of miR-21 leads to the upregulation of PTEN [105]. In GBM,* PTEN *is also targeted by miR-26a, which is upregulated by c-Myc [128]. However, copy number amplification mainly underlies the upregulation of miR-26a in GBM [95, 126, 129]. Another negative regulator of* PTEN* is miR-1908 which is upregulated in GBM relative to normal brain and low WHO grade glioma and is associated with poor prognosis [130]. The expression of* PTEN* is also repressed by miR-494-3p and miR-10a/10b, which are upregulated in GBM [131, 132]. Moreover, the high miR-10b expression levels correlate with poor prognosis in GBM patients [133]. Furthermore,* PTEN* is targeted by miR-221/222, clustered in Xp11.3, which is found to be upregulated in high relative to low WHO grade glioma [95, 134]. Additionally, the expression of the PI3K downstream effector,* Akt* (specifically* Akt1*), is regulated by miR-542-3p which is found to be downregulated in GBM [135]. miR-542-3p is negatively correlated with glioma WHO grade and is associated with poor prognosis [135].

### 6.4. miRNA Regulation of* mTOR*

Downstream of the* PI3K/Akt* pathway is mTORC1; a positive regulator of the glycolytic driver* c-Myc* is negatively regulated by the metabolic tumour suppressor AMPK which in turn is negatively regulated by miR-451 [136]. The expression of miR-451 is found to be elevated in GBM patient samples which correlates with poor prognosis [136]. miR-451 targets* CAB39*, the binding partner for the protein kinase LKB1 which phosphorylates and activates AMPK [136, 137]. The high expression levels of miR-451 are maintained by the activity of the transcription factor OCT1 [138]. This forms a positive feedback loop where low AMPK activity caused by miR-451 upregulations allows OCT1 to further drive miR-451 expression [138]. Furthermore, the expression of* mTORC1 *and* mTORC2 *is suppressed by miR-199a-3p which is downregulated in GBM compared to normal brain [139]. However, the expression of miR-199a-3p was not significantly different between low and high WHO grade glioma, suggesting that miR-199a-3p downregulation might be a key event which is tumorigenic transformation [139]. Furthermore, the mTORC2 binding partner* Rictor* is targeted by miR-34a [89, 140]. miR-34a expression, which is downregulated in GBM [88–91], is negatively correlated with* Rictor *expression, which is associated with shorter patients' survival [89].

### 6.5. miRNA Regulation of* c-Myc*

*c-Myc* is a key glycolytic driver in GBM [141]. mTORC2 positively regulates* c-Myc* expression by suppressing FoxO3a. FoxO3a enhances the expression of miR-34c which directly targets* c-Myc* [37]. mTORC2 inhibits the phosphorylation of class IIa histone deacetylases (HDACs) rendering them inactive. As such, FoxO3a remains in its acetylated inactive form. Thus, the inactivation of FoxO3a relieves the miR-34c-mediated suppression on* c-Myc* [37]. In addition to its suppression by mTORC2, the expression of* FoxO3a* is suppressed by miR-mediated mechanisms in GBM.* FoxO3a *is negatively regulated by miR-27a, which is highly expressed in GBM relative to low WHO grade glioma and normal brain and is associated with faster disease progression and shorter patient survival [84]. miR-155 is another negative regulator of* FoxO3a* which is upregulated in GBM compared to normal brain [142]. The expression of miR-155 positively correlates with glioma WHO grade and poor prognosis [143, 144].

## 7. miRNAs Regulating Aerobic Glycolysis in GBM Also Regulate Glycolytic Metabolism in Other Cancer Types

**Figure 4:**
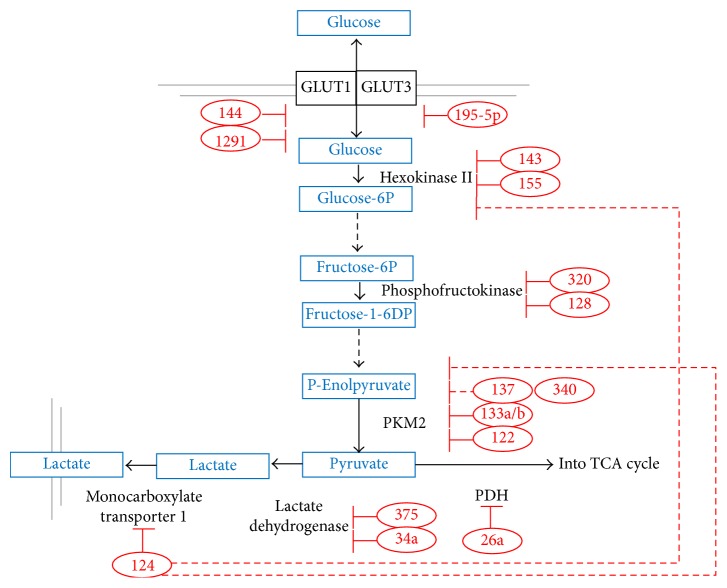
Differentially expressed miRNAs in GBM which are involved in the regulation of glycolytic metabolism in other tumours. Downregulated miRNAs are shown in red ovals. Blunt ends designate negative regulation. Double lines represent cell membrane. Dashed red lines denote indirect regulation. Dashed black lines indicate that several steps have been omitted.

Here, we attempt to link several miRNAs that regulate glycolytic metabolism in GBM, as mentioned above, to their documented glycolytic regulatory role in different cancers; these miRNAs are miR-144, miR-143/miR-155, miR-128, miR-34a, miR-340, and miR-26a as discussed below (Figure 4). miR-144, which is downregulated in GBM [87], was found to target* GLUT1 *in lung cancer [145]. The overexpression of miR-144 in lung cancer cell lines resulted in the reduction of glucose uptake and lactate production [145]. Furthermore, miR-143, which is downregulated in GBM [67, 72], has been identified as a direct regulator of* HKII* in head and neck squamous cell carcinoma (HNSCC) and in colon and lung cancer. Like in GBM, miR-143 expression is downregulated in these tumours [146–148]. Moreover, in breast cancer, miR-155 was shown to indirectly upregulate* HKII* by repressing the miR-143 transcriptional activator, CCAAT/enhancer binding protein (C/EBP) *β* [149]. miR-155 was also shown to promote* HKII *transcription by upregulating the expression of the* HKII* transcriptional activator,* STAT3* [149]. Similar to GBM, miR-155 expression is elevated in breast cancer and correlated with short survival and unfavourable clinical outcomes [144, 150]. miR-128, which is downregulated in GBM [95–99], was reported to target* PFK *in lung cancer [151]. miR-128 expression is downregulated in lung cancer and is associated with poor prognosis [151]. Another miRNA, miR-34a, which is downregulated in GBM [88–91], is also expressed at low levels in breast cancer [152, 153]. In breast cancer, miR-34a targets* LDHA* [152, 153]. In addition, in colon cancer, the PK alternative splicing proteins,* hnRNPI/hnRNAPA1/hnRNAPA2*, are targeted by miR-340, miR-124, and miR-137, which are downregulated in GBM [124, 125, 154]. In GBM, miR-137 downregulation is associated with poor prognosis [154–158]. In colon cancer, these three miRNAs, miR-340, miR-124, and miR-137, which target* hnRNPI/hnRNAPA1/hnRNAPA2*, are downregulated in order to promote the mutually exclusive alternative splicing of PK into the PKM2, which is a key metabolic adaptation in cancer [159]. Finally, miR-26a, which is upregulated in GBM [95, 126, 128, 129], is also upregulated and can target pyruvate dehydrogenase protein X component* (PDHX)* in colon cancer [160]. This would, therefore, promote glycolysis and inhibit oxidative phosphorylation (OXPHOS) by suppressing the expression of PDHX in order to block the conversion of pyruvate into acetyl coenzyme A; thereby preventing the entry of pyruvate into the citric acid cycle [160].

## 8. miRNAs Regulating Aerobic Glycolysis in Other Cancer Types Are Also Differentially Expressed in GBM

miRNAs which were reported to regulate glycolytic metabolism in different tumours are found to be differentially expressed in GBM (Figure 4). This could suggest a similar metabolic regulatory role in GBM tumours; thus, these miRNAs can serve as potential glycolytic regulators in GBM. miR-1291, for example, targets* GLUT1 *in renal cell carcinoma (RCC) and is found to be downregulated in RCC and GBM [161]. In bladder cancer, miR-195-5p, which targets* GLUT3*, is also downregulated [162]. Moreover, miR-195-5p overexpression was shown to decrease glucose uptake [162]. In GBM, miR-195-5p is downregulated and its decreased expression is associated with poor prognosis [106, 163]. In tongue squamous cell carcinoma (TSCC), the glycolytic enzyme, PKM2, is targeted by miR-133a/133b, which are downregulated in TSCC and in GBM [164–166] Moreover, miR-122, which also targets* PKM2,* is downregulated in hepatocellular carcinoma (HCC) [167] and GBM, where it correlates with shorter patients survival [168]. Moreover, the overexpression of miR-122 was shown to switch HCC cell metabolism from aerobic glycolysis to OXPHOS [167]. Furthermore, miR-124, which is downregulated in GBM [159], has been found to also be downregulated in medulloblastoma (MB) [169]. miR-124 was reported to regulate the transport of lactate into the extracellular space by targeting the lactate monocarboxylate transporter 1* (MCT1)* in MB [169]. Of interest, miR-124 was reported to target* STAT3* in GBM [170]. Since STAT3 is a transcriptional activator for* HKII* in colorectal and esophageal cancer [171, 172], miR-124 downregulation in GBM could be speculated as another miR-mediated mechanism of* HKII* upregulation. Another glycolytic enzyme, PFK, which is targeted by miR-128 as mentioned above, is also targeted by miRNA-320 in lung cancer [173]. miR-320 expression is downregulated in both lung cancer [173] and GBM [174]. A final example of differentially expressed miRNAs in GBM that regulate glycolysis in other cancers is miR-375, which targets LDHB in maxillary sinus squamous cell carcinoma (MSSCC) [175–177]. miR-375 is downregulated in MSSCC and GBM, and this associates with low survival rate [175–177]. It must be noted, however, that despite their differential expression in GBM, these miRNAs which regulate glucose metabolism in different tumours have not yet been described in relation to GBM glycolysis. Thus, carrying out functional validation studies in GBM would be necessary in order to establish such links between miRNA expression levels and their regulatory role in glucose metabolism.

## 9. Conclusion

Aerobic glycolysis is a hallmark of GBM tumours. miRNAs regulate glycolytic metabolism in GBM by directly targeting the expression of glycolytic genes and/or via the regulation of the expression of oncogenes and tumour suppressors genes in the RTKs pathways and their downstream effector pathways, such as the* PI K/Akt* pathway, which regulate glycolysis. Nevertheless, one must appreciate the complicated regulatory network that drives glycolytic metabolism and how the various individual miRNA expression changes could be interconnected with each other within the network. For example, miR-7 and let-7a modulate the expression of multiple glycolytic regulators in GBM. Furthermore, miRNAs, such as miR-34a and miR-143, which also regulate multiple glycolytic regulators in GBM, have been found to regulate glycolytic metabolism in other cancers, suggesting that such miRNAs may be regarded as universal regulators of glycolytic metabolism in cancer. On the other hand, differentially expressed miRNAs in GBM, which have not yet been linked to GBM glycolytic metabolism, were reported to have glycolytic regulatory roles in other tumours. Although the differential expression of these miRNAs in GBM could suggest a similar metabolic regulatory role in GBM, functional validation studies would be necessary before such links can be established.
